# Clinically Worsening Myasthenia-Related Respiratory Distress Notwithstanding Normal Markers of Respiratory Function

**DOI:** 10.7759/cureus.15250

**Published:** 2021-05-26

**Authors:** Hamza Ashraf, Vlad Vayzband

**Affiliations:** 1 Internal Medicine, Saint Peter’s University Hospital, New Brunswick, USA

**Keywords:** myasthenic crisis, myasthenia gravis (mg), myasthenia gravis crisis, myasthenia gravis exacerbation, vital capacity, negative inspiratory force

## Abstract

An 81-year-old female with a past medical history of myasthenia gravis presented to the Emergency Department with difficulty breathing. At presentation, the patient also complained of fatigue, diplopia, and ptosis. Vitals and laboratory tests were largely benign. The patient was diagnosed as having a myasthenia gravis exacerbation, which eventually advanced to myasthenic crisis, with the patient requiring admission to the intensive care unit and supplementation of high-flow oxygen. Throughout the course of the patient’s hospitalization, the measurements of her negative inspiratory force and vital capacity were found to be normal and unchanged despite shifting and unsteady respiratory symptoms. This uncommon case seeks to highlight the importance of complementing clinical context with the markers of respiratory function to assess the status of myasthenia-related respiratory distress.

## Introduction

Myasthenia gravis (MG) is an autoimmune disorder that affects the neuromuscular junction of skeletal muscles. The pathophysiology of MG is related to autoantibody formation against post-synaptic nicotinic acetylcholine receptors, leading to inadequate neurotransmission at the motor end plate [1]. As a result, patients experience varying and fluctuating degrees of skeletal muscle weakness that typically worsens with activity and improves with rest. Common clinical manifestations of MG include ptosis, diplopia, dysarthria, dysphagia, dyspnea, and limb or axial fatigable weakness [2]. Patients can experience transient exacerbations of these symptoms that are usually triggered by infection, stress, or improper use of medications. In some cases, the exacerbation becomes severe enough to the point where the muscles of respiration become involved, and patients require the need for either intubation or noninvasive ventilation. This is known as a myasthenic crisis [3]. Respiratory function in patients who are in myasthenia crisis is measured by both vital capacity (VC) and negative inspiratory force (NIF) [4]. Herein, we describe a case of a patient in myasthenic crisis whose respiratory symptoms worsened despite maintaining a normal VC and NIF.

## Case presentation

An 81-year-old Caucasian female with a past medical history of MG, hypertension, hyperlipidemia, depression, chronic low back pain, and rheumatoid arthritis presented to the Emergency Department due to difficulty breathing. Her difficulty breathing began one week prior to presentation, was insidious in onset, and was progressively worsening in nature. Initially, the patient became short of breath only after exerting herself, but just prior to presentation the patient became short of breath even at rest. Additionally, the patient endorsed fatigue, double vision, and droopy eyelids, all of which began at the same time as her shortness of breath. There were no complaints of orthopnea, paroxysmal nocturnal dyspnea, chest pain, palpitations, diaphoresis, nausea, vomiting, or dizziness. Vital signs on admission were within normal limits apart from an elevated blood pressure of 155/83 mm Hg. Physical examination of the patient showed an awake, alert, and oriented elderly female who was in no acute distress. Respiratory and cardiovascular examinations were completely benign, while the head, eyes, ear, nose, and throat examination was significant for bilateral ptosis that was worse on the right side as well as diplopia on both horizontal gaze and vertical gaze. The patient’s laboratory findings were insignificant as there was no anemia, no electrolyte abnormalities, and troponin, brain natriuretic peptide, and D-dimer levels were normal. The patient was found to be SARS-CoV-2 negative. A chest X-ray revealed no infiltrates and no evidence of acute cardiopulmonary disease.

The patient was diagnosed as having an MG exacerbation and was started on a five-day treatment of intravenous immunoglobulin (IVIg) alongside her home medications that consisted of pyridostigmine 120 mg every six hours and prednisone 60 mg every other day. The patient’s NIF and VC were also scheduled to be checked each shift. Despite maintaining a NIF between -55 and -65 cm H_2_O and a VC between 1 L and 1.2 L, the patient’s symptoms progressively worsened. On day 3 of hospitalization, the patient’s breathing was discovered to be extremely labored, with heavy usage of accessory muscles and the inability to fully complete sentences due to her shortness of breath. Concomitantly, a bedside pulse oximeter displayed a reading of 88% saturation of oxygen on room air. The constellation of these findings prompted an admission to the intensive care unit (ICU). In the ICU, the patient was started on high-flow nasal cannula with 30% FiO_2_, which stabilized her oxygen saturation to 95%-99%. While in the ICU, the patient’s NIF and VC remained unchanged. On day 5, after completion of IVIg treatment, the patient’s condition improved to the point where she no longer felt short of breath, was able to complete full sentences, and no longer required supplement oxygen, all of which incited downgrade from the ICU. After downgrade, the ranges of NIF and VC were again found to be unchanged from before. The patient was discharged one day after completing her IVIg treatment and was instructed to take pyridostigmine 120 mg every 6 hours and prednisone 50 mg every other day, with the addition of azathioprine 50 mg daily in hope of eventually tapering off the prednisone.

## Discussion

As the most recognized autoimmune disorder to affect the neuromuscular junction by worldwide incidence, it is estimated that between 0.3 and 2.8 per 100,000 new cases of MG are reported each year [5]. Of those newly diagnosed, women are affected twice as often as men, with a mean bimodal distribution of initial symptom onset earliest in the second to third decade of life and later in the sixth to eighth decade of life, with the latter including the mean age of onset in men [6,7]. As initial symptoms are often non-specific early in the disease course, generally encompassing fluctuating skeletal muscle weakness notability in the evening or after exertion, early detection becomes of paramount importance, especially in the elderly population [8]. Despite the relatively common and benign initial symptoms, it is estimated that one-fifth of patients may also present with myasthenic crisis, 76% of which report only generalized weakness as their inciting symptom [9,10]. The most common precipitating cause is attributed to infection, with other risk factors including surgical intervention, macrolide and fluoroquinolone antibiotics, and certain cardiac medications [11,12]. During these acute exacerbations, involvement of inspiratory and expiratory musculature leads to respiratory decline with potential need for intubation and supplemental oxygen. Involvement of initially the intercostal and accessory muscles leads to reduced expiratory capacity with subsequent diaphragmatic involvement leading to eventual cessation in inspiratory function [13].

Assessment of inspiratory function is conducted by measuring VC and NIF, with some studies showing that VC alone does not provide an adequate assessment of inspiratory status for predicting impeding respiratory collapse [13,14]. VC is used for inspiratory assessment but not in isolation, as it tests the mechanical functionality and strength during both respiratory phases. Maximal expiratory pressure should be used to assess expiratory function and becomes an important prognostic tool as it predicts the ability to clear the lungs, preventing possible infection [15]. Thus, myasthenic crisis is defined as a VC of less than 1L, an NIF of less than -30 cm H_2_O, or a maximal expiratory pressure of less than 40 cm H_2_O [16,17]. These values should be assessed within the clinical context and collectively as neither one alone provides a high predictive value. Close monitor of respiratory function and oxygenation is crucial as 66% to 90% of patients develop life-threatening respiratory failure requiring intubation [18]. Respiratory support is required when the VC drops below 20 mL/kg and/or maximal inspiratory pressure is less than -30 cm H_2_O. Additional management involves the rapid elimination of autoantibodies through plasma exchange and IVIg with adjuvant immunosuppressive therapy. Comparison between the two primary treatments has been limited, but one study has shown that the use of plasma exchange leads to quicker resolution of symptoms than IVIg but with extended use showed no significant difference [19]. Addition of immunosuppressive therapy is aimed at providing longer term protection once initial treatment wears off. Effects of immunosuppressives generally take two to three weeks [20].

## Conclusions

This study presented a case of a patient who experienced an exacerbation of MG that eventually progressed to myasthenic crisis. Despite maintaining consistently normal NIF and VC, the patient required admission to the ICU and supplemental high-flow oxygen. Completion of IVIg treatment led to the patient clinically improving. This highlights the idea that the measurements of NIF and VC have limitations in their respective prognostic and diagnostic value. Additionally, this study emphasizes the importance of clinical context as it pertains to monitoring respiratory status in patients with respiratory distress secondary to MG or myasthenic crisis.
